# Non-intubated Thoracoscopic Surgery-Tips and Tricks From Anesthesiological Aspects: A Mini Review

**DOI:** 10.3389/fsurg.2021.818456

**Published:** 2022-02-11

**Authors:** Csongor Fabo, Adam Oszlanyi, Judit Lantos, Ferenc Rarosi, Theodor Horvath, Zsanett Barta, Tibor Nemeth, Zsolt Szabo

**Affiliations:** ^1^Department of Anesthesiology and Intensive Care, University of Szeged, Szeged, Hungary; ^2^Department of Cardiac Surgery, Zala County St. Raphael Hospital, Zalaegerszeg, Hungary; ^3^Department of Neurology, Bács- Kiskun County Hospital, Kecskemét, Hungary; ^4^Department of Medical Physics and Informatics, University of Szeged, Szeged, Hungary; ^5^Department of Surgery, Masaryk University, Brno, Czechia; ^6^Department of Surgery, University of Szeged, Szeged, Hungary; ^7^Ars Medica Laser Surgery Hospital, Budapest, Hungary

**Keywords:** non-intubated thoracic surgery, VATS, spontaneous breathing, mechanical ventilation, V/Q mismatch, SVI

## Abstract

**Background:**

In the last few decades, surgical techniques have been developed in thoracic surgery, and minimally invasive strategies such as multi-and uniportal video-assisted thoracic surgery (VATS) have become more favorable even for major pulmonary resections. With this surgical evolution, the aesthetic approach has also changed, and a paradigm shift has occurred. The traditional conception of general anesthesia, muscle relaxation, and intubation has been re-evaluated, and spontaneous breathing plays a central role in our practice by performing non-intubated thoracoscopic surgeries (NITS-VATS).

**Methods:**

We performed a computerized search of the medical literature (PubMed, Google Scholar, Scopus) to identify relevant articles in non-intubated thoracoscopic surgery using the following terms [(non-intubated) OR (non-intubated) OR (awake) OR (tubeless) OR (regional anesthesia)] AND [(VATS) OR (NIVATS)], as well as their Medical Subject Headings (MeSH) terms.

**Results:**

Based on the outcomes of the reviewed literature and our practice, it seems that pathophysiological concerns can be overcome by proper surgical and anesthetic management. All risks are compensated by the advantageous physiological changes that result in better patient outcomes. With the maintenance of spontaneous breathing, the incidence of potential adverse effects of mechanical ventilation, such as ventilator-induced lung injury and consequent postoperative pulmonary complications, can be reduced. The avoidance of muscle relaxants also results in the maintenance of contraction of the dependent hemidiaphragm and lower airway pressure levels, which may lead to better ventilation-perfusion matching. These techniques can be challenging for surgeons as well as for anesthetists; hence, a good knowledge of physiological and pathophysiological changes, clear inclusion and exclusion and intraoperative conversion criteria, and good communication between team members are essential.

**Conclusion:**

NITS-VATS seems to be a feasible and safe method in selected patients with evolving importance as a part of the minimally invasive surgical and anesthetic conception and has a role in reducing perioperative complications, which is crucial in the thoracic surgical patient population.

## Introduction

In the last few decades, the focus of clinicians has shifted from invasive procedures to minimally invasive techniques, as video-assisted, preferably uniportal, thoracoscopic technique has become the gold standard for major pulmonary resections (1–4). With this surgical development, anesthesia also underwent a paradigm shift. Traditionally, video-assisted thoracic surgery (VATS) procedures require general anesthesia, muscle relaxation, and controlled ventilation with lung isolation. In recent years, the increasingly researched non-intubated techniques have become a safe and feasible alternative, with a less intense immunological response (5–7) and better oncological compliance (8). The key anesthetic consideration behind the non-intubated technique is the maintenance of spontaneous breathing with its advantageous effects on respiration during thoracoscopic or even open thoracic procedures (9–15). The thoracic surgical patient population is quite susceptible to postoperative pulmonary complications, partly arising from general anesthesia and muscle relaxation (16–18). With non-intubated techniques, the elimination of risk factors is achievable, resulting in a significantly decreased incidence of intubation-associated complications, lower postoperative VAS scores, shorter post-operative fasting period, and shorter hospital stay (19–23).

### Basic Principles of Thoracic Anesthesia

The gold standard for thoracic procedures was general anesthesia with muscle relaxation and double-lumen tube endotracheal intubation for decades. Thoracic surgery creates unique circumstances for the patients, as well as for anesthesiologists. Most of the difficulties arise from the trinity of the lateral decubitus position, open pneumothorax, and one-lung ventilation.

The lateral decubitus position in awake patients did not cause a significant change in respiratory physiology. With the induction of general anesthesia in the lateral decubitus position by decreasing the functional residual capacity (FRC), the dependent lung moves to a less favorable part of the compliance curve. A ventilation-perfusion mismatch can develop as ventilation is greater in the non-dependent lung while perfusion is still greater in the dependent lung. Due to the differences in compliance, controlled mechanical ventilation favors the non-dependent lung. These effects are augmented by the use of neuromuscular blocking agents by eliminating the physiological excursion of the diaphragm.

In most cases, one-lung ventilation is required for an optimal surgical view. During OLV, only the dependent lung is ventilated, while the perfusion of the non-ventilated lung is maintained, leading to transpulmonary right-to-left shunting. The acceptable ventilation-perfusion ratio is guaranteed by constriction of intrapulmonary arteries, a phenomenon known as hypoxic pulmonary vasoconstriction (HPV) (24–26).

All factors that inhibit the effects of HPV should be avoided. High positive airway pressures (PEEP, auto-PEEP, peak inspiratory pressure(PIP)] have antagonistic effects on HPV and divert the blood flow from the dependent areas to the non-ventilated ones (26–30). Mechanical ventilation has clear benefits in several cases, although it can also be harmful and can cause structural damage to the lungs. Regional overdistension of the lung is the key moment that can lead to ventilation-induced lung injury by inflammatory cell infiltrates, hyaline membranes, and increased capillary permeability (31, 32).

### Basic Principles and Physiology of Spontaneous Breathing

Spontaneous breathing results from a complex interaction between the chest wall, pleura, and lungs. Air flow occurs only when a pressure difference is present. Under physiological circumstances, the pressure difference between the alveolar space and the environment is the driving force of inspiration, while expiration normally occurs passively.

The diaphragm has a central role in physiological spontaneous breathing by altering the vertical dimension of the thoracic cavity, and dependent divisions of the diaphragm move with greater amplitude than the anterior (non-dependent) region, which helps to optimize the ventilation-perfusion (V/Q) matching and plays a role in preventing alveolar compression in dependent lung zones, resulting in improved ventilation. These advantages persist even in the supine position (33, 34).

It is well known that the regional distribution of ventilation is heterogeneous, and there is an increase in ventilation on the vertical axis of the lung due to the elastic properties of the lungs and the vertical gradient of pleural (and transpulmonary) pressure (35).

Moreover, one should not forget that breathing patterns, the respiratory rate, and the respiratory amplitude vary during spontaneous ventilation to meet up with the metabolic requirements that are determined by the local factors and current pressure, volume, and chemical condition of the lungs sensed by the pulmonary receptors. The main medullary rhythm generator areas were the parafacial respiratory group and the pre-Bötzinger complex (36, 37). The oscillatory activity of these regions is modified by numerous effects. Sensory afferentation occurs through both the vagal and sympathetic nerves, and airway receptors can be categorized based on electrophysiological findings. The slowly-adapting pulmonary stretch receptors sense airway pressure and the rate of change in pressure, which are insensitive to chemicals and are responsible for the Hering-Breuer reflex, which affects the frequency and amplitude of breathing. The rapidly-adapting receptors are mechanoreceptors; however, they can also be activated by chemicals. They are responsible for increased airway secretion and mucosal vasodilation. The activation of these receptors interferes with inspiration, shortens expiration, and plays a role in coughing (38, 39). Unmyelinated, chemosensitive C-fiber receptors play an important role in airway defense and airway hyper-responsiveness. Chemicals such as bradykinin, prostaglandins, hydrogen ions, and capsaicin are all able to activate these receptors, resulting in coughing and bronchoconstriction hypersecretion (38–41).

It should be mentioned that there are also several disadvantages of SB during mechanical ventilation. These disadvantages include the possibility of uncontrolled inspiratory efforts that may worsen lung injury due to volutrauma or barotrauma (42, 43) and increased heterogeneity of ventilation, leading to regional dorsal atelectrauma due to the cyclic opening and closing of small airways (43, 44), patient-ventilator asynchrony which results in patient distress (45), increased alveolo-capillary pressure gradient which leads to interstitial edema (42, 46) and impaired hemodynamics; difficulties in the feasible measurement of respiratory mechanics parameters (e.g., driving pressure) (47); and the impossibility of using NMBAs that may make endotracheal intubation and a secure airway difficult. The respiratory depression effect of major analgesics may also be a problem that requires attention.

### Brief Historical Overview of NITS

The first anecdotal report on the use of thoracic endoscopy was written in 1866 by an Irish doctor, Francis Richard Cruise, who performed thoracoscopy on an 11-year-old child with empyema. Since 1910, a Swedish physician, Hans Christian Jacobaeus, began to use thoracoscopy in patients with tuberculosis to diagnose intrathoracic diseases and performed adhesiolysis in awake patients under local anesthesia (48). Until the 1950s, increasingly complex and invasive thoracic surgical operations could be performed, mostly under local anesthesia, when double-lumen tubes were invented and introduced by Carlens and Bjork. A new era has begun, and general anesthesia with controlled, single- lung positive pressure ventilation and muscle relaxation became the gold standard in thoracic surgery. Since the late 1990s, as video-assisted thoracic surgery (VATS) has become a common procedure, less invasive anesthetic methods have been developed. To date, many tubeless, awake, and non-intubated methods are available. The common feature of these techniques is the intention to avoid intubation and general anesthesia with all their detrimental effects. There is no standard anesthesia method for NITS; it can be performed on awake subjects under locoregional anesthesia or under light or deep sedation with or without regional techniques as an adjunct (49, 50).

## Methods

We performed a computerized search of the medical literature (PubMed, Google Scholar, Scopus) to identify relevant articles in non-intubated thoracoscopic surgery using the following terms [(non-intubated) OR (non-intubated) OR (awake) OR (tubeless) OR (regional anesthesia)] AND [(VATS) OR (NIVATS)], as well as their Medical Subject Headings (MeSH) terms. Five hundred and three papers were found, and those focusing on NIVATS were excluded. Papers published before 2015, letters, and editorials were also excluded. We identified papers that provide detailed data about anesthetic management for further evaluation, and the findings of these articles are summarized below (Table 1).

**Table 1:** Characteristics and key findings from most relevant articles on non- intubated surgery.

**Author**	**Year**	**Reference no**.	**Number of patients**	**Study design**	**Context**	**Level of sedation**	**Drugs for sedation**	**Type of analgesia**	**Conversion rate**	**Key findings**
Mineo et al.	2017	(5)	55	NRC	VATS metastasectomy	Light sedation	Midazolam or remifentanil +/- propofol	ICB	NR	Less immunological and inflammatory response
Vanni et al.	2010	(6)	25	RCT	Various minor-intermediate procedures	Awake	None	TEA	NR	Lesser impact on postoperative lymphocyte responses
Tacconi et al.	2010	(7)	11	RCT	Various minor-intermediate procedures	Awake	None	TEA	NR	Decreased stress response
Furák et al.	2020	(8)	28	NRC	Uniportal VATS lobectomy	BIS: 40-60	Midazolam, fentany, propofol	ICB	NR	Improved adjuvant chemotherapy compliance and lower toxicity rates
Pompeo et al.	2004	(9)	30	RCT	Resection of small nodules	Awake	None	TEA	4 pts	Safe, feasible, better patient satisfaction, less nursing care and shorter stay
Chen et al.	2012	(12)	285	CS	Various (lobectomy, segmentectomy, wedge resection)	Ramsay III	Fentanyl, propofol	TEA	4.9%	Optimal feasibility
Wu et al.	2013	(13)	36	NRC	VATS lobectomy in elderly	Ramsay III	Fentanyl, propofol	TEA	1 pt	Optimal feasibility, faster induction, same safety
Pompeo et al.	2012	(21)	32	RCT	Non-resectional lung volume reduction surgery	Awake	None	TEA	2 pts	Shorter hospital stay
Pompeo et al.	2011	(22)	41	NRC	Non-resectional lung volume reduction surgery	Awake	None	TEA	2 pts	Better perioperative outcome, shorter hospital stay, and lower costs
Starke et al.	2020	(49)	88	CS	Various	RASS 0 to−3	Dexmedetomidine, sufentanil, propofol	TEA or PVB	6.8%	Safe and feasible for minor and major procedures
Pompeo, Mineo	2007	(51)	14	NRC	Metastasectomy	Awake	None	TEA	0	Safe and feasible. Global operating time and hospital stay were significantly shorter
Pompeo et al.	2007	(52)	21	RCT	Spontaneous pneumothorax	Awake	None	TEA	0	Feasible, shorter hospital stay, reduced costs
Katlic, Facktor	2010	(53)	384	CS	Various minor-intermediate procedures	NR	Midazolam, fentany, ketamine, propofol	LA	0	Optimal feasibility
Hung et al.	2015	(54)	238	NRC	VATS lobectomy	BIS: 40-60	Propofol, fentanyl	TEA or ICB	5.5%	Both group feasible and safe, improved haemodynamic stability and less intraoperative complications in ICB group
Li et al.	2020	(55)	57	CS	Various	BIS: 40-55	Dexmedetomidine, remifentanil, propofol	ICB	1 pt	Safe and feasible
AlGhmadi et al.	2018	(56)	31	NRC	VATS lobectomy	BIS: 40-60	Dexmedetomidine, propofol	ICB	3%	Safe and is technically feasible
Furák et al.	2020	(57)	160	CS	Various including thoracotomy	BIS: 40-60	Midazolam, fentanyl, propofol	ICB	3 pts	Major lung resections can be performed safely
Al-Abdullatief	2007	(58)	79	CS	Various including thoracotomy, sternotomy	Light sedation	Midazolam, fentanyl	TEA	1 pt	Safe and feasible even with major procedures
Moon et al.	2018	(59)	115	CS	Various	BIS: 40-60	Dexmedetomidine, propofol +/– midazolam, ketamine, fentanyl	ICB	7.8%	Non-intubated VATS is a feasible option. Older age and high BMI are risk factors for conversion
Hung et al.	2014	(60)	109	CS	Lobectomy, segmentectomy, wedge resection, tumor excision	BIS: 40-60	Propofol, fentanyl	ICB	2.8%	Technically feasible and safe

*NR, not reported; BIS, Bi-spectral index; CS, case series; ICB, Intercostal block; LA, Local anesthesia; NRC, Non-randomized comparison; RCT, Randomized-controlled trial; TEA, Thoracic epidural anesthesia*.

## Results

The pioneers of the non-intubated technique are Pompeo and Mineo (51). A non-randomized comparative study published in 2007 with 14 involved patients for single metastasis removal performed under sole thoracic epidural anesthesia with “Ventimask” to provide oxygen supplementation reported a shorter operative and global operating room time and shorter hospital stay. Another randomized controlled study from Pompeo et al. (52) with the same sole thoracic epidural catheter-based anesthesia showed shorter global operative room stay and pain control in the study group.

The first study, which included a large number of patients (353), was published in 2010 by Katlic and Factor, who performed 384 minor thoracic surgeries (drainage of empyema or lung abscess, hemothorax evacuation, pericardial fenestration, pneumothorax or chylothorax treatment, lung or mediastinal mass biopsy) between 2002 and 2009 under sole local anesthesia with individualized sedation (fentanyl-propofol-ketamine). Supplemental oxygen was provided using a face mask. They found it a safe and feasible method for minor procedures as they reported no case conversion to thoracotomy, neither was there any need for intraoperative intubation (53).

Chen et al. published a case series from Taiwan in 2012. Patients (285) underwent lobectomy, segmentectomy, or wedge resection. Anesthetic management differs from Italian methods. The common point is the insertion of a thoracic epidural catheter for optimal pain relief, but in Taiwan, mild sedation is performed with propofol target-controlled infusion (TCI) and incremental fentanyl boluses to achieve a Ramsay sedation score of III. They performed vagal nerve blockade to prevent coughing during major pulmonary resections (12, 51, 52).

Hung et al. reported a non-randomized comparison from 2015, wherein 238 patients underwent non-intubated VATS lobectomy using a thoracic epidural catheter or intercostal nerve blockade. Patients were sedated with propofol target control infusion to achieve a BIS value between 40 and 60. Supplemental oxygen was provided using a ventilation mask. Patients with intercostal nerve block showed improved hemodynamic stability and a lower incidence of intraoperative complications (54).

Li et al. investigated the feasibility of the non-intubated technique in a prospective cohort study. Fifty-seven patients who underwent lobectomy, segmentectomy, wedge resection, sympathectomy, or mediastinal tumor resection were enrolled. Anesthesia was performed by dexmedetomidine sedation followed by propofol and remifentanil TCI to reach a BIS index of 50 ± 10, with an insertion of a laryngeal mask or a nasopharyngeal airway supplemented with an intercostal and paravertebral regional block. The results suggest that the non-intubated technique is a safe and feasible alternative to general anesthesia and intubated one-lung ventilation (55). The same is stated in a retrospective comparison published by AlGhamdi et al. in 2018. They found that the non-intubated method was comparable to the intubated technique, with the trend of shorter anesthesia and operation time in the non-intubated group that was completed with a shorter hospital stay (56).

The non-intubated technique was originally designed for uniportal video-assisted surgical approach, although it is a safe and feasible option for large thoracic procedures performed with thoracotomy or even with sternotomy (57, 58).

In symbiosis with the concept of enhanced recovery after surgery (ERAS) and the development of minimally invasive surgical techniques, the concept of minimally invasive anesthesia has been established. Promotion of regional anesthetic techniques, opiate sparing helps to decrease perioperative complications and results in better pain control, reduced operation time, reduced hospital stay, and eventual better patient outcomes.

### Advantages of NITS

A recent meta-analysis from 2019 by Yu et al. investigated 27 studies that included 2,929 patients. They concluded that the NITS technique had several beneficial effects. NITS was associated with a significantly shorter hospital stay, shorter postoperative fasting time, lower costs, and shorter chest tube duration. Furthermore, NITS has stimulatory effects on cellular immunity as it is associated with increased numbers of total lymphocyte counts, T helper/T suppressor cell ratios, and natural killer (NK) lymphocyte numbers. In contrast, the aforementioned meta-analysis suggests that inflammatory and stress responses were lower among NITS subjects since white blood cells, interleukin-6, interleukin-8, C-reactive protein, procalcitonin, epinephrine, cortisol, and fibrinogen levels were lower; however, adrenocorticotropic hormone and norepinephrine levels did not differ significantly (61).

Another meta-analysis from 2020 by Wen et al. found that NITS was associated with a significantly decreased rate of postoperative complications, shorter postoperative fasting times, fewer hospital days, shorter operative and anesthesia times, and a lower risk of mortality. They concluded that NITS is a safe alternative to conventional VATS with intubated general anesthesia (19).

Furthermore, spontaneous breathing is preserved, and the diaphragm is not paralyzed, and the functional residual capacity on the dependent side is less affected during NITS (62).

Moreover, a recent study from our group by Furák et al. found oncological benefits with the NITS method, as a significantly greater proportion of non-intubated patients was able to complete the planned adjuvant chemotherapy and significantly less toxicity and neutropenia occurred among them (8).

### Risks and Concerns About NITS

To minimize the potential adverse effects and ensure patient safety inclusion and exclusion criteria needs to be clarified (Table 1). Non intubated technique is indicated for all patients who are suitable for VATS procedure with a BMI less than 30, without other exclusion criteria (Table 2).

**Table 2:** Exclusion criterias from non-intubated thoracic surgery.

**Exclusion criterias from non-intubated thoracic surgery**
Hemodynamically unstable patients
INR >1.5
Sleep apnea syndrome
Anticipated difficult airway
BMI ≥30 kg/m2
Persistent cough or high airway secretion
Elevated risk of regurgitation
Raised intracranial pressure, unable to cooperate
Procedures requiring lung isolation to protect the contralateral lung

An unsafe airway, hypoxia, and hypercapnia are the main concerns of most anesthesiologists regarding non-intubated techniques. During VATS, an open pneumothorax is induced, followed by lung collapse in the non-dependent hemithorax, and the ventilation is redirected to the contralateral side. A mediastinal shift towards the dependent lung can compromise its function, and preserved spontaneous breathing of the non-dependent hemidiaphragm can lead to the pendelluft phenomenon, resulting in hypoxia and hypercapnia due to carbon dioxide rebreathing. However, preserved diaphragmatic contractions inhibit abdominal compression against the dependent lung, counterbalancing their negative effects. Moreover, anesthetic central respiratory depression also deteriorates gas exchange. Fortunately, hypoxia can be reversed with O2 supplementation (e.g., nasal high- flow oxygen therapy or laryngeal mask airway), and CO_2_ accumulation is usually mild and well-tolerated in the perioperative period. The depth of anesthesia monitoring (e.g., the bispectral index) is helpful to avoid excessive levels of anesthesia and severe hypoventilation (19, 23, 63–65).

An extensively moving lung can make surgery difficult, necessitating conversion to intubated anesthesia. Most centers, including ours, consider high BMI (above 30 or 35) as a contraindication to NITS because obesity is associated with extensive respiratory movement with a greater motion of the mediastinum. Moon et al. found obesity to be a significant risk factor for conversion with age. Other indications for conversion to GA are summarized below (Table 3) include persistent hypoxemia, uncontrollable respiratory acidosis, unstable hemodynamic status, ineffective vagal block with a persistent cough, pleural adhesions, ineffective analgesia, panic attack, or severe intraoperative bleeding that necessitates thoracotomy (59, 60, 66, 67).

**Table 3:** Most common indication for conversion.

**Most common indications for conversion**	
**Surgical indications**	**Anesthetic indications**
Ineffective vagal block with persistent cough	**Hypoxemia**: SpO2 lower than 92% or PaO2 <60 mmHg on FiO2: 1.0, the operated (non-dependent) lung is reinflated.
Change in surgical plan (e.g. pneumonectomy, thoracotomy)	**Hypercapnia**: If PaCO2 >75 mmHg or pH <7.15, the operated lung is reinflated to eliminate CO2: if the patients breathe spontaneously via LMA low PEEP and pressure support can be applied on the circle and the lung reinflates due to this positive pressure. In case of failure conversion is necessary.
Serious diaphragm and mediastinal movements	Bleeding in the airways:
Bleeding	Ineffective analgesia
Pleural adhesions	Persistent hemodinamic instability
Large tumor size	Intraoperative airway difficulties

In anesthesia, a safe airway is of utmost importance, and anesthesiologists must have a plan in the case of an emergency. A difficult airway is a relative contraindication to NITS; however, if intraoperative conversion is needed, various methods can be used, depending on the provider's experience and preference. However, since the insertion of a double-lumen tube in the lateral decubitus position can be challenging even in experienced hands, we consider fiberoptic intubation with a single-lumen tube via a laryngeal mask airway, and bronchial blockade is a safe alternative; in our daily practice, when conversion is needed, the surgeons insert a chest tube into the wound and cover it with drapes; then, the patient is turned into the supine position. In this position, intubation can be performed easily. Our decision-to-intubation time is usually less than 2 min (57, 68).

### A New Approach in Thoracic Anesthesia: Spontaneous Ventilation Combined With Double-Lumen Tube Intubation (VATS-SVI)

The above-discussed possible disadvantages of the non-intubated techniques may discourage many specialists; however, it seems the newly-published VATS-SVI method helps in overcoming these concerns. In this method, we have all the advantages of a secured airway and the maintenance of spontaneous breathing during thoracic surgery. Mechanical one-lung ventilated time can be reduced by 76.6% with a less intense immunological response (69).

The potential short- and long- term benefits of these method remained unclear therefore further research needed to clarify them.

## Conclusion

According to the literature and our experience, non-intubated techniques are appropriate and safe procedures for minor and even major resections. With a combination of regional techniques and intravenous sedation strategies, patients successfully managed the procedures without severe hemodynamic imbalance, hypoxia, or hypercapnia. Well-thought inclusion and exclusion criteria and a clearly defined conversion protocol help to ensure patient safety. Overall, although the long-term effects of non-intubated techniques remain unclear, it seems that NITS-VATS with maintained spontaneous ventilation is a feasible and attractive alternative to GA-VATS with muscle relaxation and controlled one lung ventilation.

VATS-SVI can be a new method of ensuring maximal patient safety with reduced ventilation time and a lower incidence of complications associated with controlled mechanical ventilation. These techniques are also challenging for surgeons and anesthetists, and extensive training programs in thoracic surgery are inevitable based on experience in routine conventional techniques.

## Author Contributions

ZS, CF, AO, and TH: conception and design. JL and FR: administrative support and collection and assembly of data. All authors: provision of study materials or patients, manuscript writing, and final approval of manuscript. ZS, CF, AO, and FR: data analysis and interpretation. All authors contributed to the article and approved the submitted version.

## Conflict of Interest

The authors declare that the research was conducted in the absence of any commercial or financial relationships that could be construed as a potential conflict of interest.

## Publisher's Note

All claims expressed in this article are solely those of the authors and do not necessarily represent those of their affiliated organizations, or those of the publisher, the editors and the reviewers. Any product that may be evaluated in this article, or claim that may be made by its manufacturer, is not guaranteed or endorsed by the publisher.
